# Vivax malaria in Duffy-negative patients shows invariably low asexual parasitaemia: implication towards malaria control in Ethiopia

**DOI:** 10.1186/s12936-022-04250-2

**Published:** 2022-08-01

**Authors:** Andargie Abate, Isabelle Bouyssou, Solenne Mabilotte, Cecile Doderer-Lang, Laurent Dembele, Didier Menard, Lemu Golassa

**Affiliations:** 1 Aklilu Lemma Institute of Pathobiology, Addis Ababa University, Addis Ababa, Ethiopia; 2 College of Medicine and Health Sciences, Bahir Dar University, Bahir Dar, Ethiopia; 3 Malaria Genetics and Resistance Unit, Institut Pasteur, INSERM U1201 Paris, France; 4 ED515 Complexité du Vivant, Sorbonne Université, Paris, France; 5 Institute of Parasitology and Tropical Diseases, UR7292 Dynamics of Host-Pathogen Interactions, Federation of Translational Medicine, University of Strasbourg, Strasbourg, France; 6 Malaria Research and Training Centre (MRTC), Université des Sciences, des Techniques et des Technologies de Bamako (USTTB), Bamako, Mali; 7 Laboratory of Parasitology and Medical Mycology, Strasbourg University Hospital, Strasbourg, France

**Keywords:** Duffy antigen, Polymorphism, Asexual parasitaemia, Ethiopia

## Abstract

**Background:**

The increase in detections of *Plasmodium vivax* infection in Duffy-negative individuals in Africa has challenged the dogma establishing the unique *P. vivax* Duffy Binding Protein-Duffy antigen receptor for chemokines (PvDBP-DARC) pathway used by *P. vivax* merozoites to invade reticulocytes. Information on the impact of Duffy antigen polymorphisms on the epidemiology of *P. vivax* malaria remains elusive. The objective of this study was to determine the distribution of asexual parasitaemia of *P. vivax* according to the Duffy antigen polymorphisms in Ethiopia.

**Methods:**

DNA was extracted from dried blood spots (DBS) collected from prospectively recruited 138 *P*. *vivax-*infected patients from health centres. The identification and estimation of *P. vivax* asexual parasitaemia were performed by microscopic examination and quantitative real-time polymerase chain reaction (PCR). Duffy genotyping was conducted by DNA sequencing in a total of 138 *P.vivax* infected samples.

**Results:**

The proportion of Duffy-negatives (FY*B^ES^/FY*B^ES^) in *P. vivax* infected patients was 2.9% (4/138). Duffy genotype FY*B/FY*B^ES^ (48.6%) was the most common, followed by FY*A/FY*B^ES^ genotype (25.4%). In one patient, the FY*02 W.01/FY*02 N.01 genotype conferring a weak expression of the Fy^b^ antigen was observed. All *P.vivax* infected Duffy-negative patients showed low asexual parasitaemia (≤ 110 parasites/µL). The median *P. vivax* parasitaemia in Duffy-negative patients (53 parasites/µL) was significantly lower than those found in homozygous and heterozygous individuals (P < 0.0001).

**Conclusion:**

*Plasmodium vivax* in Duffy-negative patients shows invariably low asexual parasitaemia. This finding suggests that the pathway used by *P. vivax* to invade Duffy-negative reticulocytes is much less efficient than that used in Duffy-positives. Moreover, the low asexual parasitaemia observed in Duffy-negative individuals could constitute an ‘undetected silent reservoir', thus likely delaying the elimination of viva*x* malaria in Ethiopia.

**Supplementary Information:**

The online version contains supplementary material available at 10.1186/s12936-022-04250-2.

## Background

In 2020, the World Health Organization (WHO) estimated that malaria contributed to cause 241 million cases and 627,000 deaths [1]. Most malaria cases were caused by *Plasmodium falciparum*, while recently the prevalence and severity of *Plasmodium vivax* malaria cases has shown a sharp increment [2]. *Plasmodium vivax* is a *Plasmodium* species capable to survive even in temperate climates, which explains its wider range of geographical distribution than other species [3]. In East Shewa, Ethiopia, *P. vivax* has been responsible for 54–71.4% of malaria cases [4, 5].

Unlike *P. falciparum*, the invasion of reticulocytes by *P. vivax* requires the dual interactions between two parasite ligands (*P. vivax* Duffy Binding Protein, PvDBP and *P. vivax* reticulocyte Binding Protein 2b, PvRBP2b) and two human receptors (Duffy antigen/receptor for chemokines, DARC/CD234 and transferrin receptor 1, TfR1/CD71) [6–8].

The Duffy antigen has Fy^a^ and Fy^b^ variants differing from each other by a single nucleotide polymorphism of replacing Glycine to Aspartic acid at codon 42. Encoded by the FY gene (*ACKR1* located on chromosome 1q23.2), the Duffy glycoprotein has FY*01 (FY*A) and FY*02 (FY*B) codominant alleles. In other words, if the FY*A is inherited from one parent, the FY*B allele will be inherited from the other. Both gene products, Fy^a^ and Fy^b^ antigens are expressed on the red blood cells (RBCs). There are four main Duffy phenotypes: Fy(a + b +), Fy(a + b −), Fy(a − b +), and Fy(a − b −). The last one gives rise to Duffy-negative phenotypes [9, 10] and was thought to confer innate resistance to blood-stage infection with *P. vivax* and clinical malaria [11]. Recently, however, the widespread detection of *P. vivax* across individuals lacking Duffy antigen in many African countries [9, 12–17] including Ethiopia [13, 18, 19] further complicates the current understanding of vivax malaria epidemiology in the region.

It has been postulated that hidden transmission could be occurring in Duffy-negative populations in sub-Saharan Africa. A plausible explanation that could support this notion is the observation of Duffy-independent *P. vivax* blood-stage infection across the Duffy-negative population of Africa [20]. Most of the previous reports have given more emphasis on *P. vivax* DNA detection in Duffy-negative individuals by polymerase chain reaction (PCR) without parasite density estimation. It has been suggested that the expression of Fy^a^ and Fy^b^ antigens on the reticulocytes influenced asexual *P. vivax* parasitaemia, thereby modulating susceptibility to clinical vivax malaria [21]. For instance, in Papua New Guinea, it has been shown that Duffy heterozygous (FY*A/FY*B^ES^) individuals had lower *P. vivax* parasitaemia than those individuals with homozygous (FY*A/FY*A) [22]. To date, the association of clinical *P. vivax* with Duffy antigen is contradicting. For instance, genotypes FY*A/FY*A, as compared to FY*B/FY*B, were reported in the increasing susceptibility of individuals and the severity of *P. vivax* infection [23]. In contrary, some studies have shown that FY*B/FY*B genotypes were highly susceptible to *P. vivax* malaria [21, 24–26].

In Ethiopia, data regarding the epidemiological features of vivax malaria in relation with duffy blood group are scarce [13, 18, 19], while an admixture of people with varying Duffy antigen polymorphism co-exists and *P. vivax* transmission is frequent. This provides the opportunity to study distribution and effect of Duffy antigen polymorphisms on the asexual *P. vivax* parasitaemia. This study was, therefore, initiated to explore this question in the admixed populations of Duffy-positive and Duffy-negative individuals in East Shewa, Ethiopia.

## Methods

### Study area and population

**Fig. 1:**
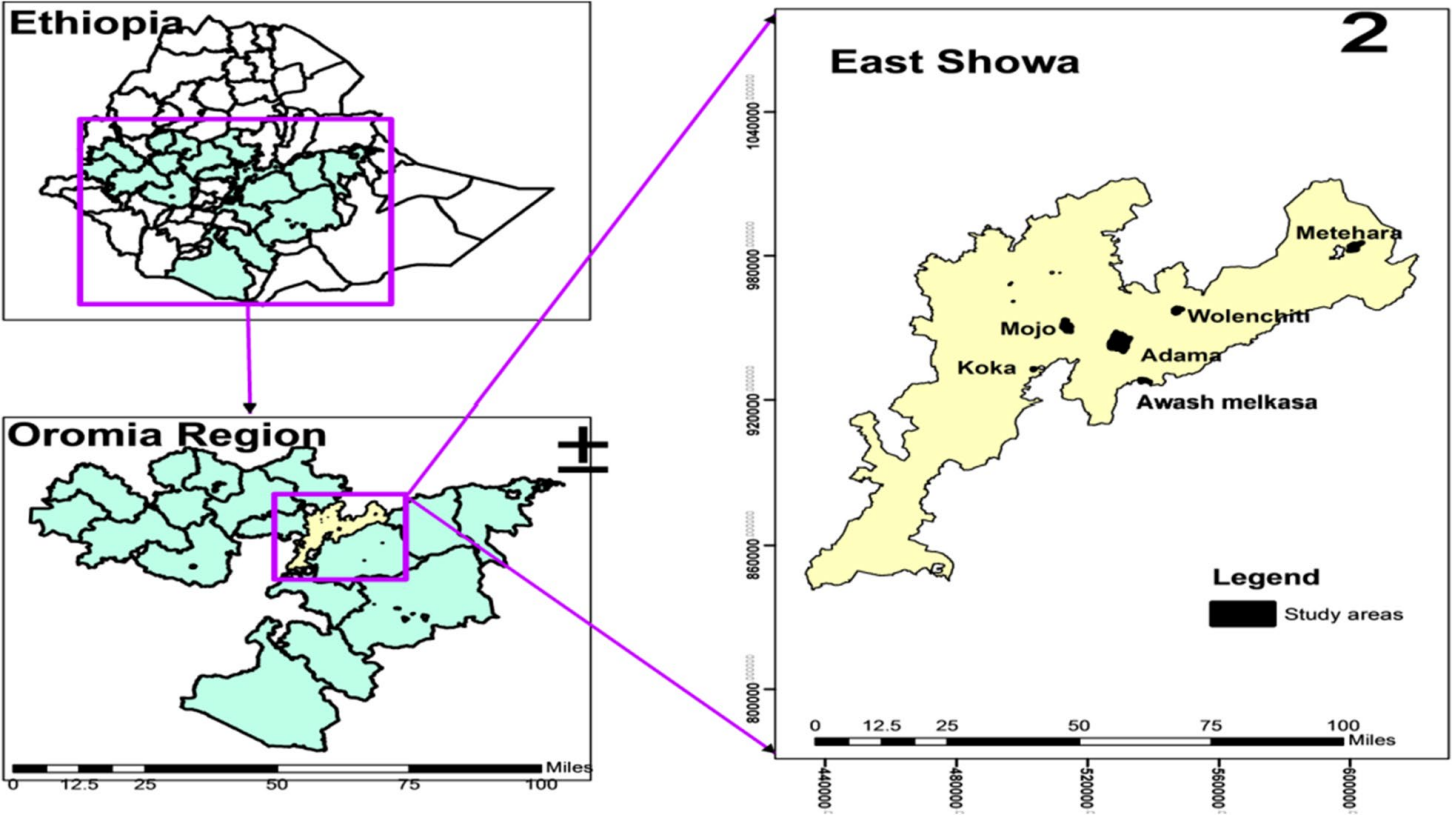
Map showing the geographical distribution of study sites in East Shewa, Ethiopia

The study was conducted in Adama City Administration, and its surroundings located 100 km southeast of Addis Ababa, the capital city of Ethiopia from October 2019 up to January 2021 (Fig. 1). Adama City Administration is presented on 1623 m above sea level, 20.5 °C average annual temperature and 808 mm annual rainfalls. *Plasmodium vivax* is reported as the predominant malaria-causing species [4, 5] in East Shewa. A total of 151 individuals were prospectively recruited from all health centres. The study populations were those patients with fever seeking health care in public health institutions at Adama City Administratin (malaria diagnostic centre, and 7 health centres), and surroundings (Awash Melkasa, Koka, Metehara, Mojo and Welenchity health centres) in East Shewa Zone. All patients known to have uncomplicated *P. vivax* malaria by microscopy were included when possible. Those with *P. falciparum* and a recent history of antimalarial drugs were excluded from the current study.

### Blood sample collection

Clinical *P. vivax* blood samples were collected from patients seeking malaria diagnosis and treatment at health centres. Finger-prick blood samples were collected to determine *Plasmodium* infection. Both thin and thick blood films were prepared in a single slide and labeled with the identification number of participants and date of preparation. The thin smear was fixed with methanol, and both thick and thin blood smears were stained with Giemsa according to the protocol [27]. Each slide was examined independently by two experienced microscopists who were blinded to each other’s result under a microscope with 100 × oil immersion. Slides with discordant results were read by a third reader blindly, and the majority results were taken as final. If a *P. vivax* infection was confirmed by microscopy examination, two to four spots of blood, near to  ~ 60 μl each spot, were collected on Whatman 3 MM filter paper for dried blood spots (DBS) for each participant, dried, and stored at room temperature until use.

### DNA extraction

DBS were cut into small pieces with scissors and transferred into 2 mL microtubes. Genomic DNA was extracted using QIAamp DNA Mini blood kit according to the manufacturer’s protocol with some modifications. Briefly, 1 mL of PBS (1X)/saponin (0.5%), 180 µL of buffer ATL, 20 µL proteinase K, 200 µL of Buffer AL, 200 µL of ethanol (96–100%), 500 µL of Buffer AW1, 500 µL of Buffer AW2, and 200 µL of elution buffer (AE buffer) were added to each tube. The extraction was performed using the recommended incubation temperature and time with manufacturers’ centrifugation rate. Finally, the 1.5 mL Eppendorf tubes containing the extracted DNA were stored at − 20 °C until use.

### Identification of *Plasmodium* species by real-time PCR

*Plasmodium* detection was performed by real-time PCR amplification of species-specific segments of the *cytochrome b* using the protocol described previously [28]. The assay was conducted to confirm and quantify *P. vivax* and mixed infection with *P. falciparum* since only these species were detected in the microscopic examination. The following nucleotide primers were used: forward-5′-TGCTACAGGTGCATCTCTTGTATTC-3′, and reverse-5′-ATTTGTCCCCAAGGTAAAACG-3′ specific for *P. vivax*, and forward-5′-ATGGATATCTGGATTGATTTTATTTATGA-3′, reverse-5′-TCCTCCACATATCCAAATTACTGC-3′ specific for *P. falciparum*. The PCR amplifications using CFX96™ Real-Time PCR detection system (Bio-Rad Laboratories Ltd) were carried out following 10 μL of molecular water, 4μL of Evagreen HRM Mix (5x) (Sotis biodyne), 0.5 μL of each primer, and 5 μL of DNA template with a final volume of 20 μL. The thermal cycling programs were 94 °C for 15 min (minutes) followed by 20 cycles of 30 s (seconds) at 94 °C, 58 °C for 1 min, 72 °C for one and a half min and 72 °C for 10 min followed by a cooling step of 20 °C. Negative (water) and positive (*P. vivax* DNA from blood sample with parasite density estimated ~ 8000 parasites/µL and *P. falciparum* DNA at 5 ng/µL) control samples were used in each PCR run. For each sample positive for *P. vivax*, the qPCR Ct value was estimated based on a threshold defined at 200.

### Estimating parasite density from qPCR Ct value

The parasite densities per µL were estimated using qPCR for all 138 samples. However, to observe the correlation between qPCR ct values with microscopy results, the parasite densities per µL of fourteen randomly selected samples were determined on Giemsa-stained blood films. Blood films were read for 30 min under oil-immersion microscope by three experienced technicians (approximately 20,000 red blood cells). The correlation between qPCR Ct values (triplicate) with microscopy results (triplicate) was estimated using scatter diagrams and correlation coefficients (automatic weighted regression).

### Duffy genotyping

Duffy genotypes were determined by PCR amplification from *P. vivax* human isolates since the DBS samples also contained human DNA. The primers for genotyping were forward-5′-GAGGCTTGTGCAGGCAGT-3′ and reverse-5′-CAAACAGCAGGGGAAATGAG-3′ specific for GATA-1 transcription factor binding site of the FY gene (− 33 T → C), and forward-5′-CCCTCAATTCCCAGGAGACT-3′ and reverse-5′-GCTGAGCCATACCAGAC ACA-3′ specific for exon 2 of the DARC gene (125 G → A) (Sigma Aldrich, France). The PCR was conducted in a 50 μL total PCR volume using 33 μL molecular water, 1 μL of each primer, 10 μL of 5X HOT blend Taq polymerase (Sotis biodyne), and 5 μL of DNA template, as previously described with minor modifications [29]. The PCR conditions were first denaturation at 94 °C for 15 min followed by 40 cycles at 94 °C for 20 s, 60 °C for 20 s, 72 °C for 1 min, and final elongation at 72 °C for 10 min. The PCR products were purified using Agencourt AMPure XP bead-based purification prior to sequencing. The purified PCR products were sent to Biofidal (France) for Sanger sequencing. The sequenced nucleotides were analyzed, using CLC Genomics Workbench 22.0 (Qiagen, Germany) software, on both strands. Sequences produced from the present study were aligned with their respective reference sequences.

### Statistical analysis

All statistical analyses were performed with MedCalc software (version 20.014) and Statistical Package for Social Sciences (SPSS) version 25. Descriptive statistics were used to describe the characteristics of study participants, frequencies of Duffy genotypes, phenotypes, and other related variables using figures and tables. Outlier detection was used to detect anomalous observations in sample data by the Tukey test [30]. The outliers were categorized as ‘outside’ or ‘far out’ values. An outside value was defined as a value that is smaller than the lower quartile minus 1.5 times the interquartile range, or larger than the upper quartile plus 1.5 times the interquartile range. A far-out value was defined as a value that is smaller than the lower quartile minus 3 times the interquartile range, or larger than the upper quartile plus 3 times the interquartile range.

The regression analysis was used to define the relationship between asexual *P. vivax* parasitaemia estimated by microscopy and qPCR Ct value (defined by the best-fitting regression model), and then to deduce the parasitaemia from qPCR Ct values. The relationship was then confirmed with the paired t-test, intraclass correlation coefficient (ICC), and Passing-Bablok regression (bootstrap method). The independent non-parametric Mann–Whitney test was used to determine the differences between the median *P. vivax* parasitaemia related to Duffy antigen polymorphism with 95% confidence interval (CI).

The violin plot [31] was used to present the distribution of asexual *P. vivax* parasitaemia by Duffy antigen polymorphism. A P-value < 0.05 was considered statistically significant.

## Results

### Characteristics of the study population

Out of the total 151 recruited patients, 11 were *P. falciparum* positive(confirmed by two microscopists), and 2 were negative (confirmed by PCR). Thus, 138 symptomatic *P. vivax* infected patients were enrolled in this study; of which 135 were *P. vivax* single infections and the remaining three were mixed infections (*P. vivax* and *P. falciparum*) as determined by microscopy and real-time PCR. Thirty seven percent of the study participants were in the age group of 15–24 years old. The highest number (37%) of study participants were from Adama City Administration. The majority (66.7%) of study participants were males and were recruited during the data collection year of 2020 (67.4%) (Table 1).

**Table 1:** Characteristics of study participants in East Shewa, Ethiopia

Variables	Category	Number (%)
Sex	Male	92 (66.7)
	Female	46 (33.3)
Age	≤ 14	16 (11.6)
	15–24	51 (37.0)
	25–34	38 (27.5)
	≥ 35	33 (23.9)
Data collection period	2019	31 (22.5)
	2020	93 (67.4)
	2021	14 (10.1)
Data collection centres/health institutions	Adama City Administration	51 (37.0)
	Awash Melkasa	7 (5.1)
	Koka	3 (2.2)
	Metehara	29 (21.0)
	Mojo	18 (13.0)
	Welenchity	30 (21.7)

### *Plasmodium vivax* parasitaemia deduced by qPCR

*Plasmodium vivax* DNA was detected by qPCR amplification in all tested samples (N = 138). The median (range) Ct value was 19.07 (12.92–36.62). The outlier detection revealed 12 (8.7%) outside values in the sample set (Additional file 1: Fig. S1). The correlation between qPCR Ct values with microscopy results from the 14 randomly slected blood samples showed by the best*-*fitting regression model was estimated with the exponential curve as regression equation (Additional file 1: Fig. S2, Table S1). The ICC value indicated excellent consistency between microscopy and qPCR Ct values (ICC = 0.927; 95% CI 0.865–0.961). The paired *t*-test indicated that the estimated (from microscopy) and deduced (from qPCR Ct values) parasitaemia were not significantly different (mean difference 188.5 parasites/µL, 95% CI − 593.2 to 970.2, P = 0.629). The Passing–Bablok regression did not present evidence of a systematic or proportional bias. The estimations for the Intercept (95% CI) were − 9.92 (− 163.02 to 37.33), for the Slope (95% CI) 0.93 (0.84–1.14) and for the Spearman rank correlation coefficient (95% CI) 0.87 (0.78–0.93, P < 0.00001) (Additional file 1: Fig. S3).

The deduced median (range) *P. vivax* asexual parasitaemia of the 138 infected study participants was 4804(38–26,278) parasites/µL. We detected 12 (8.7%) samples with far-out values (≤ 110 parasites/µL) (Additional file 1: Fig. S4).

### Duffy blood group genotyping

Duffy genotyping was successfully performed on 138 patients as shown in Table 2. The proportion of Duffy-negatives (FY*B^ES^/FY*B^ES^) in *P. vivax* infected patients was 2.9% (4/138). The duffy negatives were detected from Adama, Welenchity, Koka and Metehara (one from each site). Duffy genotype FY*B/FY*B^ES^ (48.6%) was the most common followed by FY*A/FY*B^ES^ genotype (25.4%). The FY*02 W.01/FY*02 N.01 genotype confering a weak expression of the Fy^b^ antigen was observed in one patient. The FY*02 was the most frequently observed allele (65.9%) followed by FY*01 (42.8%). The most common (54.3%) phenotype detected in the study was Fy(a-b +) (Table 2).

**Table 2:** Genotypes and phenotypes frequencies of Duffy blood group among *P. vivax* infected patients in East Shewa, Ethiopia

DbSNP	rs2814778	rs12075	rs34599082	rs13962	Predicted genotype	Predicted antigen	Phenotype	No. (%)
Nucleotide	c.1-67^a^	c.125^b^	c.265^c^	c.298^d^				
Region	5′UTR	Exon 2	Exon 2	Exon 2				
SNP (amino acid)	C	A(Asp42)	C(Arg89)	G(Ala100)	FY*B^ES^/FY*B^ES^ or FY*02 N.01/FY*02 N.01	Fy^es^/Fy^es^	Fy(a − b −)	4 (2.9)
	T	G(Gly42)	C(Arg89)	G(Ala100)	FY*A/FY*A or FY*01/FY*01	Fy^a^	Fy(a + b −)	7 (5.1)
		A(Asp42)	C(Arg89)	G(Ala100)	FY*B/FY*B or FY*02/FY*02	Fy^b^	Fy(a − b +)	6 (4.3)
		G(Gly42)/A(Asp42)	C(Arg89)	G(Ala100)	FY*A/FY*B or FY*01/FY*02	Fy^a^/Fy^b^	Fy(a + b +)	17 (12.3)
	C/T	A(Asp42)	C(Arg89)	G(Ala100)	FY*B/FY*B^ES^ or FY*02/FY*02 N.01	Fy^b^/Fy^es^	Fy(a − b +)	67 (48.6)
		A(Asp42)	C(Arg89)	A(Thr100)	FY*02/FY*02 N.01	Fy^b^/Fy^es^	Fy(a − b +)	1 (0.7)
		A(Asp42)	C(Arg89)/T(Cys89)	G(Ala100)/A(Thr100)	FY*B^ES^/FY*X or FY*02 W.01/FY*02 N.01	Fy^x^/Fy^es^	Fy(a − b +)	1 (0.7)
		G(Gly42)/A(Asp42)	C(Arg89)	G(Ala100)	FY*A/FY*B^ES^ or FY*01/FY*02 N.01	Fy^a^/Fy^es^	Fy(a + b −)	35 (25.4)

*NB*: the nucleotide position (dbSNP) is based on NCBI data, *UTR*: untranslated region

^a^This polymorphism prevents expression of Fy^b^ antigen in red blood cells

^b^This polymorphism predicts the expression of the Fy^a^ and Fy^b^ antigens

^c,d^These polymorphisms determine weak expression of the Fy^b^ antigen

### Association between Duffy phenotypes and deduced *P. vivax* asexual parasitaemia

To determine whether and how Duffy phenotypes influence the asexual parasitaemia in *P. vivax* patients, this study analysed the relationship between the deduced parasite densities and the Duffy antigens distribution (deduced from Duffy genotypes). The median (range) *P. vivax* asexual parasitaemia of the 138 infected study participants by Duffy phenotypes are presented in the Table 3.

**Table 3:** Association between Duffy antigen polymorphism and *P. vivax* asexual parasitaemia in patients from East Shewa, Ethiopia

Phenotype	Predicted antigen	Deduced parasite density (parasites/µL)			
		No. of sample	Range	Median (95% CI)	Interquartile range
Fy(a + b +)	Fy^a^/Fy^b^	17	39–26,278	4584 (2824–6122)	2543–6637
Fy(a + b −)	Fy^a^	7	3905–8750	4725 (4198–8714)	4530–7899
	Fy^a^/Fy^es^	35	75–18,247	6042 (4126–7425)	3479–7937
Fy(a − b +)	Fy^b^	6	2811–7053	3893 (2811–6785)	2811–5781
	Fy^b^/Fy^es^	68	39–20,100	4987 (3903–6178)	2693–7880
	Fy^x^/Fy^es^	1	5943	5943	–
Fy(a − b −)	Fy^es^/Fy^es^	4	38–76	53	39–72
All		138	38–26,278	4804 (4242–5781)	2811–7663

**Fig. 2:**
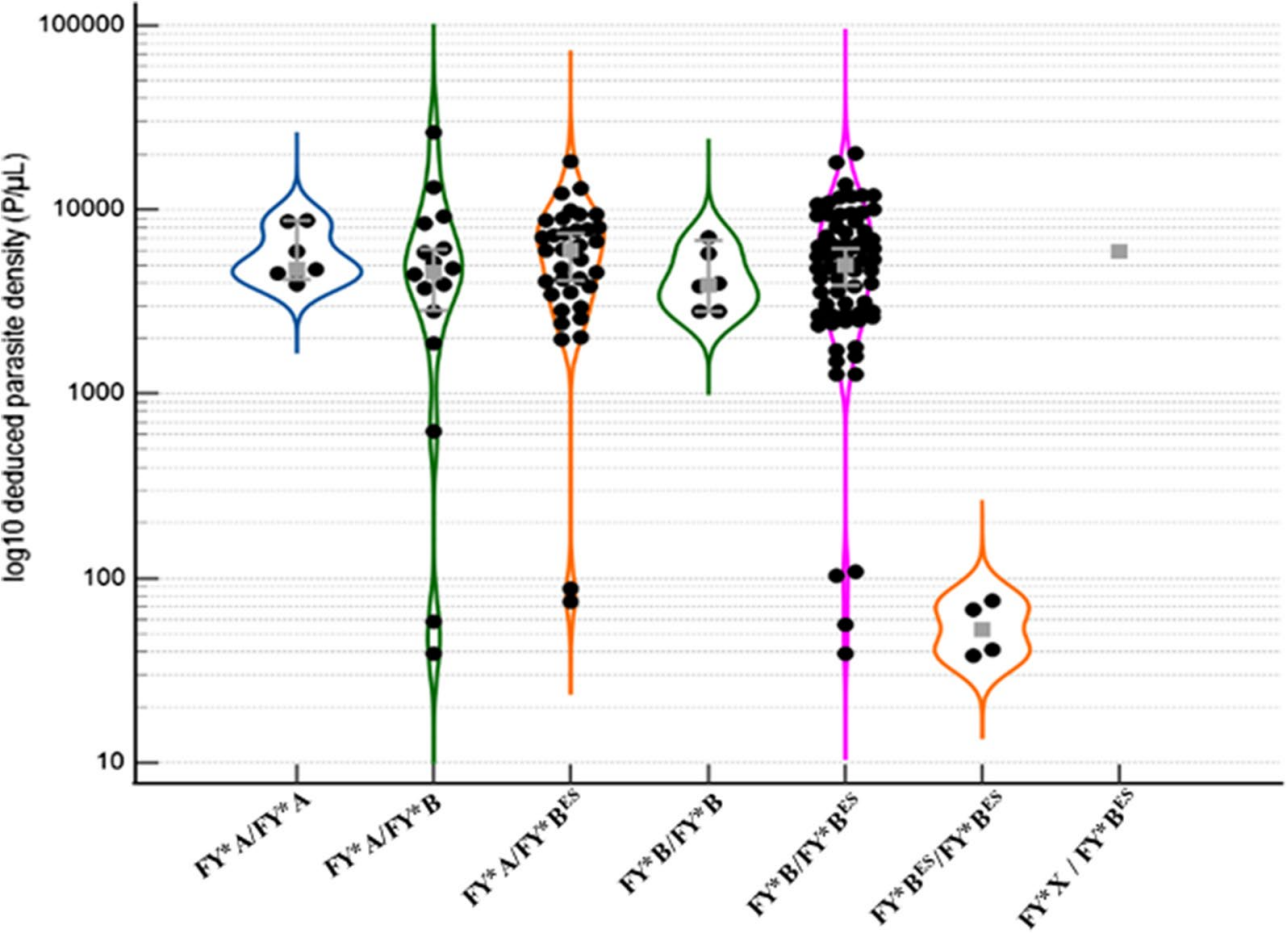
Distribution (violin plot) of *P. vivax* asexual parasitemia by Duffy genotypes detected in patients in East Shewa, Ethiopia

*Plasmodium vivax* infected Duffy-negative patients (FY*B^ES^/FY*B^ES^) showed a regularly low asexual parasitaemia (median, 53 parasites/µL) significantly lower (100-fold) than homogzygous (FY*A/FY*B, FY*A/FY*A or FY*B/FY*B) and heterozygous (FY*A/FY*B^ES^, FY*B/FY*B^ES^ or FY*B^ES^/FY*X) individuals (P < 0.0001). No significant difference in asexual *P. vivax* parasitaemia was observed between homozygous and heterozygous Duffy-positive individuals. Interestingly, four of the Duffy-negative patients were classified in the ≤ 110 parasites/µL group, while the proportions of patients carrying other Duffy phenotypes who had ≤ 110 parasites/µL were lower (5.7% [for FY*A/FY*B^ES^]) and (3.0% [for FY*B/FY*B^ES^]) (Fig. 2).

### Discussion

Since the first discovery of Duffy blood groups in the 1950s [32], there have been many investigations focusing on its global distribution [33], its role in blood transfusion [34], and for *P*. *vivax* invasion pathway into red blood cells [35]. Later, the innate resistance of humans to *P*. *vivax* infections has been attributed to Duffy blood group polymorphisms [26]; however it is unclear whether Duffy blood group polymorphism confers either only partial or complete resistance against *P. vivax*. This is because of the detection of *P. vivax* infections [36] among the previously refractory Duffy antigen-negative red blood cells [11] as frequently observed in Africa over the last decade [13, 14, 19].

In the current study, *P. vivax* infection in Duffy-negative individuals was identified. However, the proportion (2.9%) of *P. vivax*-infected patients with Duffy-negatives was lower than previous studies conducted at different parts of Ethiopia (10.5–11.9%) [13]. Here, a larger number of FY*B/FY*B^ES^ (Fy^b^/Fy^es^) and FY*A/FY*B^ES^ (Fy^a^/Fy^es^) carriers were observed among *P. vivax* infected patients in agreement with the previous study [19], indicating frequent heterozygous Duffy negativity of the study participants. This was in contrast with the predominant record of homogenous FY*B^ES^ in most African ethnic groups [10, 21], but confirmed the variation in the frequency of the Duffy genotypes across different populations. For instance, FY*B/FY*B was frequently observed in Eastern, while FY*B/FY*B^ES^ was in Southwestern Ethiopia [19]. A higher frequency of FY*B allele carriers was observed compared with the FY*A carriers. A consistent finding is that the probability of having the FY*B allele has increased in Africa [9, 19], in contrast to Asian countries where FY*A was the most frequent variant [25].

Phenotypically, the current results showed that the most common Duffy phenotype was Fy(a − b +) followed by Fy(a + b −) with a frequency of 54.3% and 30.5%, respectively, consistent with previous observations in neighbouring countries, such as Sudan [9]. Although all patients in the current study were clinical, the finding may suggest that individuals with Fy(a + b −) have a relatively low susceptibility to clinical *P. vivax* malaria compared to Fy(a − b +) due to diminishing effect of Fy(a + b −) on the binding of Duffy binding protein (PvDBP) at the erythrocyte surface, as it has been shown in Brazil [21]. The relatively more frequent observation of Fy(a − b +) phenotypes among *P. vivax* infected patients compared to healthy individuals in Sudan [9] also provided potential evidence for an association between Fy(a − b +) and its susceptibility to clinical malaria.

The FY*X (FY*02 W.01) allele encoded the weakly expressed Fy^b^ antigen due to the reduced amount of Duffy protein caused by the mutation at position 89 (Arg > Cys) [37, 38]. Surprisingly, we observed for the first time that FY*X/FY*B^ES^(FY*02 W.01/FY*02 N.01) genotype, which is not common in Africa [10], was detected from one *P. vivax* infected patient. The asexual *P. vivax* parasitaemia (5943 parasites/µL) detected in this patient was, however, similar to those found in homozygous and heterozygous Duffy-positives.

To date, it is well known that the asexual parasitaemia in patients with *P. vivax* malaria is influenced by several factors such as immunity, age, delay in seeking treatment, self-treatment behaviour before presentation, and other variety of host and parasite factors eventhough no significant differences were observed in the current study. Here, this study aimed at exploring an additional factor which is the effect of Duffy antigen polymorphisms that can also modulate asexual parasitaemia in *P.vivax* patients. The current study observed that heterozygous Duffy-positive individuals had similar range of parasite density with homozygous, as supported by previous findings in Ethiopia [13] and Thailand [39], but contrary to reports from Papua New Guinea suggesting that *P. vivax* parasitaemia was significantly lower in heterozygous than homozygous [22]. Particularly, Michon et al. showed the advantages of being heterozygous to reduce Duffy binding protein adherence to erythrocytes connecting with resistance against clinical *P. vivax* malaria [40].

However, the current study confirmed that the parasite density of *P. vivax* was significantly lower in Duffy-negative individuals than the Duffy-positives in line with previous studies elsewhere [9, 13, 22]. From this study, all Duffy-negative patients had parasitaemia ≤ 110 parasites/µL (median 53 parasites/µL), while Duffy-positive patients (homozygous or heterozygous) had an average parasitaemia between 1000 and 5000 parasites/µL. These data suggest two important features regarding vivax malaria epidemiology in Africa.

First, the very low parasite density observed in Duffy-negative patients might provide better estimates of the silent *P. vivax* parasites reservoir that may remain undetected by widely used microscopic examination although all *P. vivax* in Duffy negatives in the current study were detected by microscopy. It is important to note that the current frequent observation of vivax malaria among Duffy negative Africans might be due to the use of a more sensitive molecular tools. This suggests that *P. vivax* malaria was likely missed in Duffy-negative patients in endemic settings such as Africa where malaria diagnosis is routinely made by microscopy. This was supported by the previous findings which indicated that only four of the twenty *P. vivax* infections were microscopic-positive in Duffy-negative patients in other parts of Ethiopia [13]. This, therefore, further pointed out that *P. vivax* might be maintained in the Duffy-negative populations without clinical symptoms [41], and with the difficulty of microscopic examination [13], but could be transmitted between hosts [42].

This hypothesis is further substantiated by the fact that several studies noticed the high probability of transmission of *P. vivax* between hosts from submicroscopic individuals [43–45]. However, further study is needed to determine the relative contribution of Duffy antigen polymorphism in the transmissibility of *P.vivax* gametocytes to mosquitoes. The observation of *P.vivax* among Duffy-negative individuals, thus, might further complicate the poor understanding of the exact frequency of vivax malaria in Ethiopia and thereby across Africa where both Duffy-negatives and -positives co-exist.

Second, the very low parasite density found in Duffy-negative patients strongly suggests that the invasion pathway(s) used by *P. vivax* merozoites to invade Duffy-negative reticulocytes is (are) far less efficient compared to the classical pathway involving the interaction between the PvDBP and DARC in Duffy positive individuals. One hypothesis that is warranted to be envisioned is to consider that reticulocytes (or less mature erythroid cells from the bone marrow) of Duffy-negative subjects, which were thought to be devoid of the Duffy protein, express transitorily and weakly this protein at the course of erythroid cell differentiation. This hypothesis had already been explored by Dechavanne et al. [46], but needs to be confirmed and validated. However, this cannot reject the alternative hypothesis that *P. vivax* pathway used to invade Duffy negative reticulocytes is based on not very effective alternative interactions between unknown parasite ligand and reticulocytes receptors.

The limitation of this study includes failure to well describe the association of factors other than Duffy antigen polymorphism which could affect parasitaemia of *P.vivax* because of small samples. In addition, the number of Duffy negatives was too small for making firm conclusion regarding predictors of the parasite density.

In conclusion, the current study described the emerging perspectives on the relationship of Duffy antigen polymorphisms with parasite density of *P. vivax*. The complex relationships between hosts and parasites have clinical implications including the risk of being a silent reservoir, and thus needs introducing specific and sensitive diagnostic methods in areas where *P. vivax* malaria is endemic. Further investigations using longitudinal study and large samples (both clinical and community) from different parts of Ethiopia are needed to know the exact distribution of Duffy antigen and how Duffy antigen polymorphisms impact and shape the *P. vivax* malaria epidemiology in Ethiopia.

## Supplementary Information

**Additional file 1: Table S1.** Summarized statistics of the regression equations (weighted least-squares regression) tested as the best-fitting regression model between *P. vivax* parasitemia estimated by microscopy and qPCR Ct values. **Figure S1.** Distribution of the qPCR Ct values of the 138 *P. vivax* blood samples collected from *P.vivax* infected patients, 2019–2021. The median (range) Ct value was 19.07 (12.92–36.62). The Outlier detection using the Tukey test revealed 12 (8.7%) outside values in the sample set (red dots). **Figure S2.** Scatter diagram of the best-fitting regression model (exponential curve as regression equation) between qPCR Ct values and microscopy results from the 14 blood samples. **Figure S3.** Passing–Bablok regression: scatter diagram and regression line of *P. vivax* parasitemia defined by microscopy and parasitemia deduced from qPCR Ct values. The Intercept (95% CI) were – 9.92 (− 163.02 to 37.33); the Slope (95% CI) 0.93 (0.84–1.14) and the Spearman rank correlation coefficient (95% CI) 0.87 (0.78–0.93, P < 10–5). **Figure S4.** Distribution of the *P. vivax* parasitemia deduced from qPCR Ct values. The median (range) P. vivax asexual parasitemia of the 138 infected study participants was 4804 (38–26,278) parasites/µL. The Tukey test (Outlier detection) detected 12 (8.7%) samples with far-out values (below 110 parasites/µL).

## Data Availability

The data that support the findings of this study are available from the corresponding author on reasonable request. All relevant data are within the manuscript.

## Abbreviations

DARC: Dufy antigen/receptor for chemokines
RBCs: Red blood cells
SNP: Single nucleotide polymorphism
PCR: Polymerase chain reaction
PvDBP: *Plasmodium vivax* Dufy binding protein
PvRBP2b: *Plasmodium vivax* Reticulocyte Binding Protein 2b
TfR1: Transferrin receptor 1
WHO: World Health Organization
