# Supplementary material for: Vivax malaria in Duffy-negative patients shows invariably low asexual parasitaemia: implication towards malaria control in Ethiopia
Source: Malar J. 2022 Aug 1;21:230. doi: 10.1186/s12936-022-04250-2 (PMC9341100; doi:10.1186/s12936-022-04250-2)
Supplement: Supplementary file 1 — Additional file 1: Table S1. Summarized statistics of the regression equations (weighted least-squares regression) tested as the best-fitting regression model between P. vivax parasitemia estimated by microscopy and qPCR Ct values. Figure S1. Distribution of the qPCR Ct values of the 138 P. vivax blood samples collected from P.vivax infected patients, 2019–2021. The median (range) Ct value was 19.07 (12.92–36.62). The Outlier detection using the Tukey test revealed 12 (8.7%) outside values in the sample set (red dots). Figure S2. Scatter diagram of the best-fitting regression model (exponential curve as regression equation) between qPCR Ct values and microscopy results from the 14 blood samples. Figure S3. Passing–Bablok regression: scatter diagram and regression line of P. vivax parasitemia defined by microscopy and parasitemia deduced from qPCR Ct values. The Intercept (95% CI) were – 9.92 (− 163.02 to 37.33); the Slope (95% CI) 0.93 (0.84–1.14) and the Spearman rank correlation coefficient (95% CI) 0.87 (0.78–0.93, P < 10–5). Figure S4. Distribution of the P. vivax parasitemia deduced from qPCR Ct values. The median (range) P. vivax asexual parasitemia of the 138 infected study participants was 4804 (38–26,278) parasites/µL. The Tukey test (Outlier detection) detected 12 (8.7%) samples with far-out values (below 110 parasites/µL). [file 12936_2022_4250_MOESM1_ESM.doc]

**Sublementary materials**

**Table S1. Summarized statistics of the regression equations (weighted least-squares regression) tested as the best-fitting regression model between *P. vivax* parasitemia estimated by microscopy and qPCR Ct values**

| Regression equation | straight line | Logarithmic curve | **Exponential curve** | Geometric curve | Quadratic regression |
| --- | --- | --- | --- | --- | --- |
|  | y = a x + b | y = a + b log(x) | **log(y) = a + b x** | log(y) = a + b log(x) | y = a + b x + c x^2^ |
| Sample size | 42 | 42 | **42** | 42 | 42 |
| Coefficient of corelation R² | 0.68 | 0.72 | **0.94** | 0.92 | 0.83 |
| Residual standard deviation | 1.18 | 1.17 | **1.32** | 1.21 | 1.21 |
| The equation of the regression curve | y = 15850 - 473 x | y = 42426 - 27972 log(x) | **log(y) = 5.97 - 0.12 x** | log(y) = 11.97 - 6.58 log(x) | y = 41735 - 2695 x + 43 x^2^ |
| Analysis of variance (*P* value) | <0.0001 | <0.0001 | .<0.0001 | <0.0001 | <0.0001 |

**Coefficient of determination R^2^**: this is the proportion of the variation in P. vivax parasitemia explained by the regression model and is a measure of the goodness of fit of the model. It can range from 0 to 1; **Residual standard deviation**: the standard deviation of the residuals (residuals = differences between observed and predicted values); **Analysis of variance**: the analysis of variance table divides the total variation in the P. vivax parasitaemiainto two components, If the significance level for the F-test is small (less than 0.05), then the hypothesis that there is no (linear) relationship can be rejected.

**
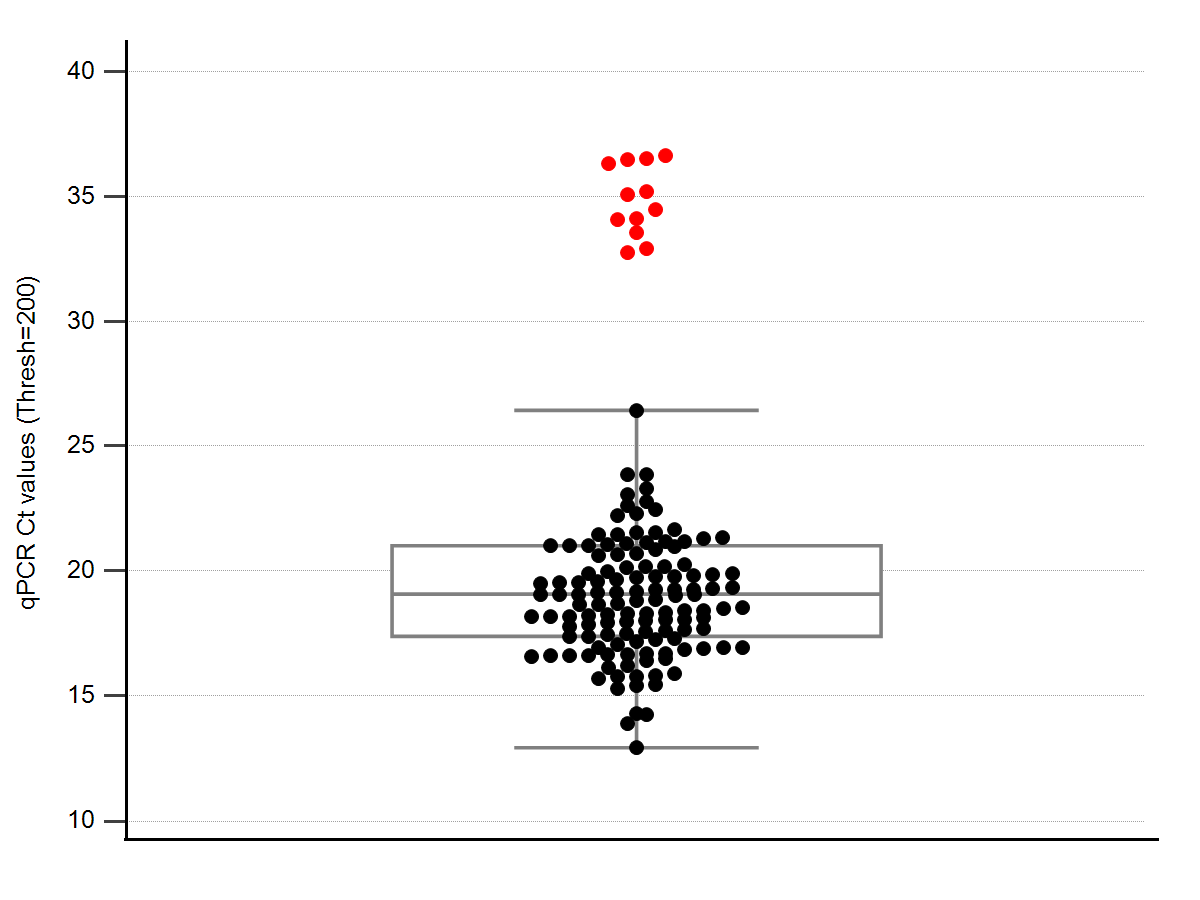
**

**Figure S1.** Distribution of the qPCR Ct values of the 138 *P. vivax* blood samples collected from *P.vivax* infected patients, 2019-2021.

The median (range) Ct value was 19.07 (12.92-36.62). The Outlier detection using the Tukey test revealed 12 (8.7%) outside values in the sample set (red dots)


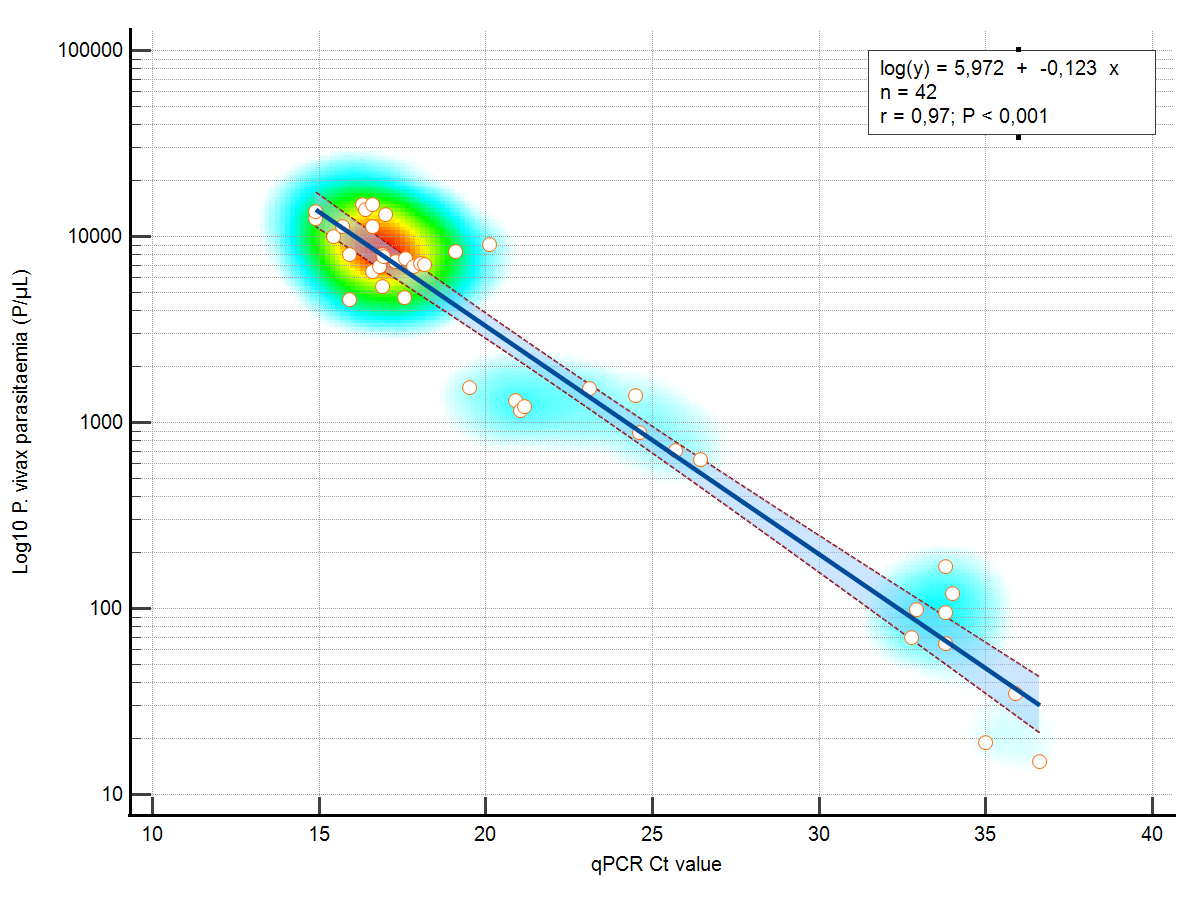


**Figure S2.** Scatter diagram of the best-fitting regression model (exponential curve as regression equation) between qPCR Ct values and microscopy results from the 14 blood samples.


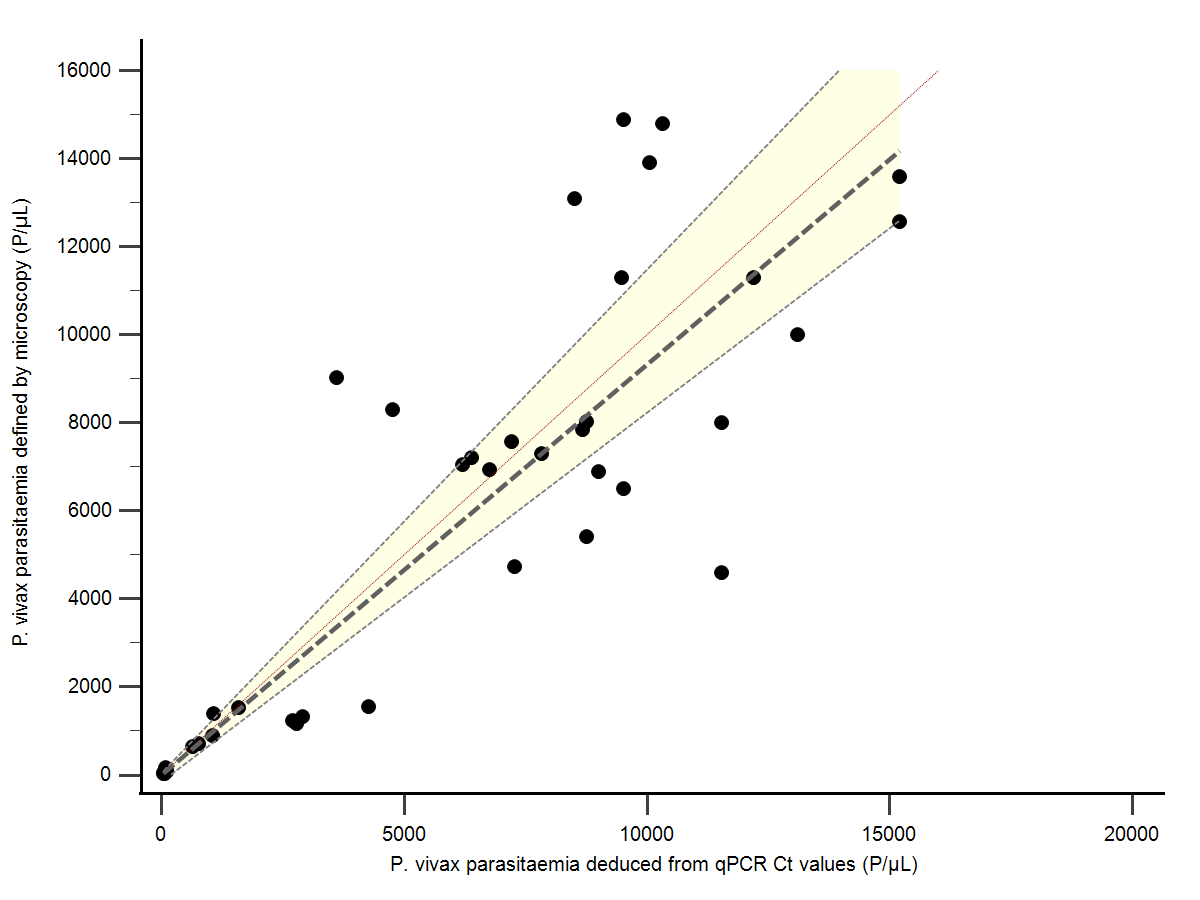


**Figure S3.**Passing–Bablok regression: Scatter diagram and regression line of *P. vivax* parasitemia defined by microscopy and parasitemia deduced from qPCR Ct values.

The Intercept (95% CI) were -9,92 (-163.02 to 37.33); the Slope (95% CI) 0,93 (0.84-1.14) and the Spearman rank correlation coefficient (95% CI) 0,87 (0.78-0.93, p <10-5)


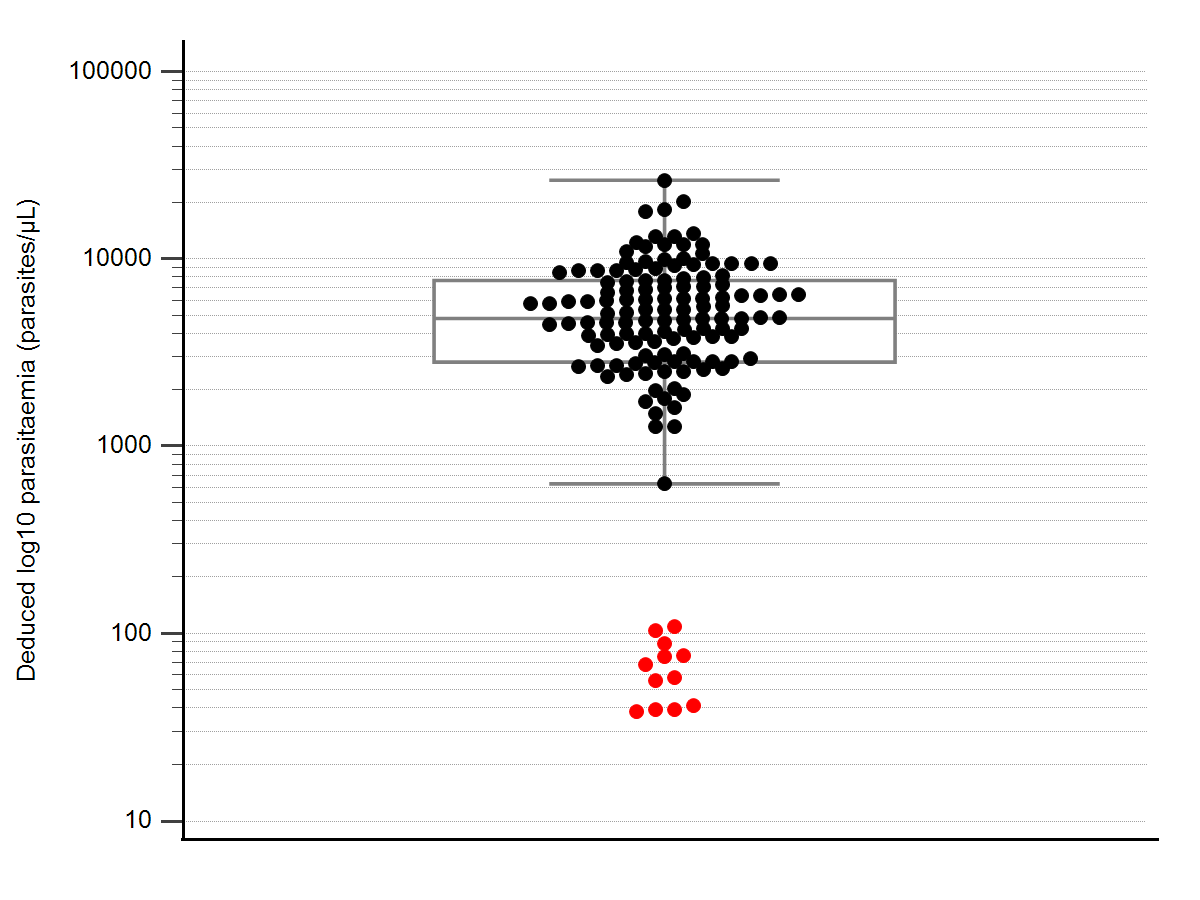


**Figure S4.**Distribution of the *P. vivax* parasitemia deduced from qPCR Ct values.

The median (range) P. vivax asexual parasitemia of the 138 infected study participants was 4,804 (38-26,278) parasites/µL. The Tukey test (Outlier detection) detected 12 (8.7%) samples with far-out values (below 110 parasites/µL).
